# Association Between Abscess Size, Inflammatory Markers, and the Need for Drainage in Renal Abscesses

**DOI:** 10.3390/diseases14050160

**Published:** 2026-04-30

**Authors:** Dragoș Puia, Marius Ivănuță, Ovidiu Daniel Bîcă, Nicolae Stoican, Mihaela Corlade-Andrei, Bogdan Doroftei, Cătălin Pricop

**Affiliations:** 1 Grigore T. Popa University of Medicine and Pharmacy Iasi, 700115 Iasi, Romania; dragos-puia@umfiasi.ro (D.P.); marius.ivanuta@umfiasi.ro (M.I.); catalin.pricop@umfiasi.ro (C.P.); 2 Department of Urology, “Dr. C.I. Parhon” Clinical Hospital, 700503 Iasi, Romania; 3 Center for Morphological and Spectroscopic Analysis of Urinary Stones” Michel Daudon”, 700503 Iasi, Romania; 4 Department of Urology, “Sf. Ioan cel Nou” County Emergency Hospital, 720033 Suceava, Romania; 5 Emergency Care Department, “Sf. Spiridon” County University Emergency Hospital, 700111 Iasi, Romania; 6 Origyn Fertility Center, Palace Street No. 3C, 700032 Iasi, Romania; 7 Clinical Hospital of Obstetrics and Gynecology “Cuza Voda”, Cuza Voda Street No. 34, 700038 Iasi, Romania

**Keywords:** renal abscess, inflammatory markers, percutaneous drainage, antibiotic resistance

## Abstract

Background: Renal abscesses represent a serious complication of urinary tract infections, with management decisions often being guided by abscess size and clinical parameters. However, there is no universally accepted size threshold for intervention, and the role of inflammatory markers such as white blood cell count (WBC) and C-reactive protein (CRP) in guiding treatment remains uncertain. We aimed to evaluate the relationship between abscess size, inflammatory markers, and the need for drainage in patients with renal abscesses treated in a tertiary urology clinic. Methods: A retrospective analysis was conducted on 103 adult patients diagnosed with renal abscesses between 2020 and 2025. Patients were categorized into two groups based on abscess size: Group A (<50 mm) and Group B (50 mm). Results: The cohort included 59 females and 44 males, with a mean age of 60.5 years. Computed tomography was used for diagnosis in 55.3% of cases. The most common comorbidities were hypertension (46.6%) and diabetes mellitus (40.8%). Microbiological cultures most frequently identified *Escherichia coli* (38.3%) and *Klebsiella* spp. (21.7%). Antibiotic resistance was highest to ampicillin (79.5%), while amikacin (5.8%) and piperacillin/tazobactam (6.2%) showed the lowest resistance rates. Conservative antibiotic therapy was effective in 43 patients (42.7%), whereas 60 patients (58.3%) required percutaneous drainage. Abscess size was associated with invasive intervention, with 88.1% of drained abscesses measuring ≥50 mm compared to 9.1% in the conservatively managed group (*p* < 0.001). Patients with larger abscesses had significantly lower haemoglobin levels (*p* = 0.003), while no significant differences were observed in WBC or CRP levels. Conclusions: Abscess size was associated with the need for drainage, supporting its role in clinical decision-making. In contrast, inflammatory markers such as WBC and CRP showed limited value in predicting the need for intervention in this cohort. These findings should be interpreted in the context of the retrospective design.

## 1. Introduction

Renal abscess represents a localized suppurative infection involving the renal parenchyma or perinephric space, most commonly arising as a complication of ascending urinary tract infection and, less frequently, via hematogenous dissemination. Although relatively uncommon compared to other urinary tract infections, renal abscesses remain clinically significant due to their potential for severe complications, including sepsis, renal function impairment, and, in advanced cases, the need for nephrectomy. Early recognition is essential, yet diagnosis is often delayed because clinical manifestations are nonspecific, typically including fever, flank pain, and general malaise, which overlap with other renal or systemic infections [1].

The occurrence and severity of renal abscesses are strongly influenced by underlying patient factors. Comorbidities such as diabetes mellitus, urinary tract obstruction, nephrolithiasis, and immunocompromised states predispose patients to more severe infections and may alter both clinical presentation and therapeutic response. From a microbiological perspective, Gram-negative bacilli, particularly *Escherichia coli* and *Klebsiella pneumoniae*, are most frequently implicated, reflecting their predominant role in urinary tract infections. However, variability in microbial profiles and increasing antimicrobial resistance further complicate management strategies and highlight the need for individualized therapeutic approaches [2,3].

Imaging plays a central role in both diagnosis and therapeutic decision-making. Computed tomography (CT) and ultrasonography are considered standard imaging modalities in clinical practice, allowing for not only detection of abscesses but also characterization of their size, location, and complexity. CT is generally regarded as the reference method due to its higher sensitivity and ability to delineate anatomical details, while ultrasonography remains widely used due to its accessibility and lack of radiation exposure. These imaging techniques have significantly improved diagnostic accuracy and have facilitated earlier intervention, contributing to better clinical outcomes [4,5].

The management of renal abscesses requires a balance between conservative and invasive strategies. While small abscesses may be resolved with antibiotic therapy alone, larger or more complex collections often require percutaneous or surgical drainage. In current clinical practice, abscess size is widely used as a key parameter guiding this decision. However, there is no universally accepted size threshold for intervention, and treatment strategies remain heterogeneous across institutions. In addition to imaging findings, clinical evolution under antibiotic therapy is frequently considered, further contributing to variability in management approaches [6,7,8].

Inflammatory markers such as white blood cell count (WBC) and C-reactive protein (CRP) are routinely used in the evaluation of infectious diseases and are commonly elevated in patients with renal abscesses. Despite their widespread use, their role in predicting disease severity, abscess size, or the need for drainage remains uncertain. It is unclear whether these markers provide clinically meaningful information beyond imaging parameters, particularly in guiding interventional decisions [9,10].

Despite advances in imaging and management, there is no universally accepted abscess size threshold for drainage, and the independent predictive value of inflammatory markers such as WBC and CRP remains unclear. Clarifying whether these markers add value beyond imaging is clinically relevant, particularly in borderline cases.

Therefore, the aim of this study was to evaluate the relationship between abscess size, inflammatory markers, and the need for drainage in patients with renal abscesses in the context of clinical practice, while also describing the associated microbiological profile and antibiotic resistance patterns.

## 2. Materials and Methods

### 2.1. Study Design

The study was conducted as a retrospective analysis of all adult patients (≥18 years) diagnosed with renal abscesses at the Urology Clinic of “Dr. C. I. Parhon” Clinical Hospital, Iași, Romania, between January 2020 and December 2025. The hospital is a tertiary referral center, and all consecutive cases during this period were included. Data were obtained from electronic medical records and had been collected as part of routine clinical care. Ethics approval was obtained from the institutional Ethics Committee (Approval No. 8271/1 September 2025) prior to data analysis, and the study was conducted in accordance with the Declaration of Helsinki.

Patients were included if they had imaging-confirmed renal abscess (CT or ultrasound) and complete clinical, laboratory, and imaging data available. We excluded cases of uncomplicated acute pyelonephritis without abscess formation, postoperative or non-infectious collections, and patients with incomplete data.

Data were extracted retrospectively from electronic medical records. We recorded demographic characteristics (age, sex), comorbidities (diabetes mellitus, hypertension, nephrolithiasis), laboratory values at admission (WBC, haemoglobin, platelet count, BUN, serum creatinine, and C-reactive protein), microbiological results, imaging findings, treatment approach, and clinical outcomes.

Antibiotic resistance rates were calculated for each antimicrobial agent as the proportion of isolates resistant to that specific antibiotic.

### 2.2. Imaging, Abscess Measurement, and Treatment Approach

Diagnosis was established using CT, US, or both, depending on the clinical context.

Laboratory parameters were measured in the hospital’s central laboratory using standardized automated analysers, in accordance with institutional protocols. Laboratory parameters were recorded at the time of admission.

Imaging data were obtained using ultrasonography and/or contrast-enhanced computed tomography, performed by experienced radiologists following standard diagnostic procedures. CT was considered the reference method when available due to its higher sensitivity and accuracy in assessing abscess characteristics.

Abscess size was defined as the maximum diameter measured on imaging. When both CT and US were available, we used the CT measurement, given its higher accuracy. In patients evaluated only by ultrasonography, the largest reported dimension was used.

For patients with multifocal abscesses, we used the size of the largest lesion for analysis, as this was considered the most relevant parameter for treatment decisions. Patients were divided into two groups based on abscess size (<50 mm and 50 mm).

The 50 mm threshold was selected based on previously published studies suggesting that larger abscesses are less likely to respond to antibiotic therapy alone and more frequently require drainage.

All patients received empirical antibiotic therapy at admission, which was later adjusted according to urine culture results.

The decision to perform percutaneous drainage was primarily based on abscess size at presentation, but also on clinical evolution, including persistence of symptoms, presence of sepsis, and lack of response to antibiotic therapy within 48–72 h. In selected cases, drainage was performed for smaller abscesses when clinical deterioration or lack of improvement was observed.

No strictly standardized institutional protocol was applied, and treatment decisions were ultimately based on the clinical judgment of the attending physician.

Treatment decisions were not based on a strictly standardized protocol and were ultimately made at the discretion of the attending physician. Drainage catheters were maintained until output decreased and imaging confirmed resolution. Treatment success was defined as resolution of the abscess without the need for further invasive procedures.

Nephrectomy was reserved for selected patients with severe disease, most often in the presence of extensive renal parenchymal destruction or persistent infection despite drainage and appropriate antibiotic therapy.

### 2.3. Statistical Analysis

Continuous variables were expressed as mean ± standard deviation. The normality of continuous variables was assessed using the Shapiro–Wilk test. Continuous variables were approximately normally distributed, and comparisons were performed using Student’s *t*-test. Categorical variables were analysed using the chi-square test. Cases with missing data were excluded from the analyses.

Spearman’s rank correlation coefficient was used to assess the relationship between abscess size, inflammatory markers (WBC, CRP), haemoglobin, and serum creatinine.

Univariate logistic regression analysis was performed to evaluate the association between individual variables and the need for drainage.

Receiver operating characteristic (ROC) curves were used to assess the ability of abscess size, WBC, and CRP to predict the need for drainage.

A multivariable logistic regression model was performed using the need for drainage (yes/no) as the dependent variable and abscess size, WBC, and CRP as independent variables.

The proportion of patients requiring drainage was compared between abscess size groups (<50 mm vs. 50 mm), and the corresponding risk ratio (RR) with 95% CI was calculated.

A *p*-value < 0.05 was considered statistically significant. Statistical analysis was performed using SPSS version 26.0.

## 3. Results

A total of 103 patients were included in the analysis, comprising 59 females and 44 males, with a mean age of 60.5 ± 16.4 years. Abscess size was assessed using computed tomography (CT) alone in 57 cases (55.3%), a combination of CT and ultrasonography (US) in 27 cases (26.2%), and ultrasonography alone in 19 cases (18.4%). The most common comorbidities were hypertension (46.6%), diabetes mellitus (40.8%), and nephrolithiasis (30.1%).

Conservative antibiotic therapy alone was successful in 43 patients (42.7%), whereas 60 patients (58.3%) required percutaneous drainage. Among those undergoing drainage, 18 patients (30.0%) subsequently required nephrectomy. Patients requiring nephrectomy had a significantly larger mean abscess diameter compared to the rest of the cohort (101.1 ± 27.5 mm, *p* < 0.00001).

As shown in Table 1, the need for drainage was associated with abscess size. Among patients who underwent drainage, 88.1% had abscesses 50 mm, compared to only 9.1% in the conservatively treated group (*p* < 0.001). No statistically significant differences were observed between groups in terms of WBC, CRP levels, age, or comorbidities. However, haemoglobin levels were significantly lower in patients requiring drainage (*p* = 0.024), while length of hospital stay was similar between groups.

Given the association between abscess size and treatment approach, we further evaluated the risk of requiring drainage according to abscess size category. Patients with abscesses 50 mm had a higher risk of requiring drainage compared with those with abscesses < 50 mm (RR = 6.35, 95% CI 3.19–12.59, *p* < 0.001), as shown in Table 2.

To further explore the clinical differences associated with abscess size, patients were stratified into two groups (<50 mm and 50 mm), and their characteristics are presented in Table 3. The mean diameter was 25.2 ± 8.7 mm in the <50 mm group and 90.0 ± 33.0 mm in the 50 mm group (*p* < 0.0001). No statistically significant differences were identified between groups in terms of WBC, CRP, age, or most comorbidities. However, patients with larger abscesses had significantly lower haemoglobin levels (*p* = 0.003).

Microbiological findings and antibiotic resistance patterns are summarized in Table 4. The most frequently isolated pathogens were *Escherichia coli* (38.3%) and *Klebsiella* spp. (21.7%), followed by *Enterococcus* spp. and *Proteus* spp. (13.3% each). Antibiotic resistance was highest for ampicillin, while the lowest resistance rates were observed for amikacin and piperacillin/tazobactam.

As shown in Table 5, no significant associations were observed between abscess size and inflammatory markers, with negligible correlations for both WBC and CRP. WBC and CRP were moderately correlated, while abscess size showed a weak negative correlation with haemoglobin. No significant correlation was found with serum creatinine, suggesting a limited relationship between inflammatory markers and abscess size in this cohort.

As shown in Table 6, univariate logistic regression analysis identified abscess size as a significant predictor of the need for drainage. Haemoglobin showed a borderline inverse association with the likelihood of intervention. In contrast, no significant associations were observed for WBC, CRP, age, or serum creatinine. These findings further support the central role of abscess size, while suggesting a limited contribution of inflammatory markers in this setting.

**Figure 1:**
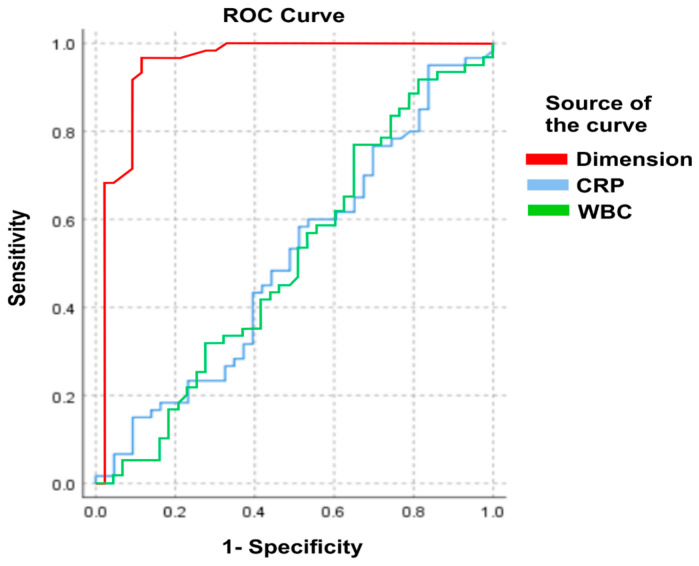
ROC analysis of the abscess dimension, WBC, and CRP, and treatment method.

ROC analysis demonstrated that abscess size had excellent discriminatory ability for predicting the need for drainage (AUC = 0.948), whereas WBC (AUC = 0.502) and CRP (AUC = 0.507) showed no meaningful discriminatory value. The corresponding ROC curves are presented in Figure 1.

To assess whether inflammatory markers provided additional predictive information beyond abscess size alone, logistic regression models were constructed, as presented in Table 7.

Model 1 includes abscess size only (univariate model). Model 2 is a multivariable model adjusted for abscess size, WBC, and CRP. The dependent variable was the need for drainage. Values are presented as OR with 95% CI.

The predictive performance of the models is shown in Table 8. As can be seen, the addition of inflammatory markers resulted in only a minimal and non-significant improvement in discrimination (AUC 0.945 vs. 0.949; ΔAUC = 0.004, *p* = 0.74).

## 4. Discussion

Renal abscess remains a clinically significant condition due to its potential for severe complications and the need for timely diagnosis and management. Its occurrence is strongly influenced by underlying comorbidities such as diabetes mellitus, urinary tract obstruction, and nephrolithiasis, which were also frequently observed in our cohort [2,3]. Although relatively uncommon compared to other urinary tract infections, renal abscesses require a structured diagnostic and therapeutic approach due to their potential for progression to sepsis or renal impairment [2,4].

In our study, abscess size was associated with the need for drainage. Most patients requiring intervention had abscesses larger than 50 mm, reflecting their role in clinical decision-making. This finding is consistent with previous studies suggesting that larger abscesses are less likely to respond to antibiotic therapy alone and more frequently require percutaneous drainage [11,12,13,14]. However, as highlighted in the literature, a universally accepted size threshold is still lacking, and management strategies remain heterogeneous across institutions [15,16].

In contrast, inflammatory markers did not show a significant association with either abscess size or the need for drainage. Although leucocytosis and elevated CRP are commonly reported in patients with renal abscesses [15,16], reflecting the systemic inflammatory response, our results suggest that these markers have limited value in guiding interventional decisions when only baseline measurements are considered. In the acute clinical setting, management decisions are often based on initial laboratory values; however, their ability to discriminate between patients requiring drainage and those managed conservatively appears limited. This is supported by the poor discriminatory performance observed in ROC analysis and by the lack of improvement in regression models after including WBC and CRP.

An important aspect in interpreting these findings is the relationship between abscess size and clinical decision-making. In our cohort, drainage was primarily performed based on abscess size, with clinical evolution under antibiotic therapy playing a secondary role. This introduces a degree of circularity, as abscess size functions both as a biological parameter and as a determinant of treatment. As a result, the observed association between abscess size and the need for drainage may partly reflect existing treatment patterns rather than a fully independent predictive effect.

In this context, the need for drainage, although clinically relevant, remains a physician-driven decision and may not fully reflect disease severity. Other endpoints, such as failure of conservative treatment or septic progression, could provide additional insight and should be considered in future studies.

From a diagnostic perspective, imaging remains central in the evaluation of renal abscesses. Ultrasonography is widely used as a first-line modality due to its accessibility, but its sensitivity is limited, particularly for smaller lesions, and results may be operator-dependent [16,17,18,19]. In contrast, CT provides superior anatomical detail and is considered the reference method for diagnosis and treatment planning [20,21]. In our study, a proportion of abscesses were assessed by ultrasonography alone, which may have introduced variability in size estimation, especially for lesions near the 50 mm threshold.

Microbiologically, our findings are in line with the existing literature, with *Escherichia coli* and *Klebsiella pneumoniae* being the most frequently isolated pathogens [16,22]. The high resistance rates observed for commonly used antibiotics, particularly ampicillin, further emphasize the importance of culture-guided therapy. Pus culture remains the most reliable method for pathogen identification, as urine cultures may be negative in cases where the abscess does not communicate with the collecting system [19,23]. While abscess size is frequently used in clinical practice when considering drainage, an additional contribution of our study is the characterization of the local microbiological profile and antibiotic resistance patterns, which may have practical implications for empirical treatment.

The management of renal abscesses requires a balance between conservative and invasive approaches. While small abscesses may be successfully treated with antibiotics alone, larger or non-resolving collections often require percutaneous drainage [11,12,13,14]. In addition to size, clinical response to antibiotic therapy is an important determinant, and lack of improvement within 48–72 h should prompt consideration of intervention [24]. Surgical drainage or nephrectomy is generally reserved for complex or refractory cases, particularly in the presence of extensive parenchymal destruction or severe sepsis [25,26,27,28]. The relatively high rate of nephrectomy in our cohort likely reflects the severity of disease at presentation, with surgery being required in patients who failed to respond to drainage and antibiotic therapy or had extensive renal parenchymal destruction.

Recent studies have explored the potential role of novel biomarkers, such as circulating microRNAs, in improving risk stratification in infectious diseases. These biomarkers may better reflect the host inflammatory response and disease severity. However, they are not currently part of routine clinical practice. In this context, our findings suggest that commonly used inflammatory markers, such as WBC and CRP, provide limited information for guiding interventional decisions, highlighting the need for more reliable biomarkers in future studies [29].

Lower haemoglobin levels observed in patients with larger abscesses or requiring drainage may reflect the impact of systemic inflammation on red blood cell homeostasis, as inflammatory states associated with infection can impair erythropoiesis and alter iron metabolism. Pro-inflammatory cytokines such as IL-6 play a central role by increasing hepcidin expression, which limits iron export from macrophages and enterocytes, leading to reduced iron availability for haemoglobin synthesis despite adequate or increased iron stores [30,31].

In this context, anemia is likely multifactorial, resulting from the combined effects of suppressed erythropoiesis, iron-restricted erythropoiesis, and increased red blood cell clearance that can occur during infection and inflammation. These mechanisms, together with the complex interplay between nutritional status, comorbidities, and infectious burden, may contribute to lower haemoglobin levels and reflect a higher overall disease burden rather than an isolated laboratory abnormality [32,33].

Several limitations of this study should be acknowledged. First, its retrospective design introduces potential selection and information bias. Second, abscess size was not assessed uniformly, as some patients were evaluated using ultrasonography alone, which may be less accurate than CT. Third, treatment decisions were not standardized and were largely influenced by abscess size and clinical judgment, which may have affected the observed associations. In this context, abscess size was used both as a factor guiding treatment and as a variable in the analysis.

The multivariable model included a limited number of variables and did not account for several clinically relevant factors, such as urinary obstruction, stone burden, multifocal disease, hemodynamic status, or early response to antibiotic therapy, which may also influence the decision to perform drainage. Finally, the analysis was limited to commonly used inflammatory markers, and other biomarkers that are potentially associated with disease severity were not evaluated. Despite these limitations, this study provides real-world data from a tertiary center and directly compares imaging parameters and inflammatory markers in the context of clinical decision-making. By integrating clinical, laboratory, and imaging data, our findings highlight the close relationship between abscess size and drainage decisions within our clinical practice.

## 5. Conclusions

In our study, abscess size was associated with the need for drainage, with larger abscesses being significantly more likely to require intervention, consistent with current clinical practice.

In contrast, inflammatory markers such as WBC and CRP did not differ significantly between groups and did not improve the prediction of drainage. Although these markers reflect the presence of infection, their role in guiding interventional decisions appears limited.

These findings suggest that imaging, particularly abscess size, remains a central element in clinical decision-making. In borderline cases, clinical evolution under antibiotic therapy may be more informative than isolated laboratory values. However, these findings should be interpreted with caution, as abscess size also influenced the decision to perform drainage in our cohort.

## Figures and Tables

**Table 1:** Patients’ characteristics according to treatment type.

Parameter	Overall (n = 103)	Conservative (n = 43)	Drainage (n = 60)	*p*
Age (years)	60.53 (±16.3).	58.98 (17.7)	61.65 (15.4)	0.20
Gender F/M	59/44	26/17	33/27	0.58
Rural/Urban, n	62/41	23/20	39/21	0.23
Length of stay (days)	10.45 (±5.8)	10.3 (±5.5)	11.2 (±4.5)	0.41
WBC (/mmc)	13,893.3 (±6971.4)	14,330.9 (±8316.3)	13,579.7 (±5877.7)	0.29
Hb (g/dL)	10.65 (±2.1)	11.12 (±2.1)	10.28 (±2.1)	0.024
CRP (mg/dL)	142.14 (±100.6)	142.35 (±104.2)	141.85 (±99.1)	0.49
Serum creatinine (mg/dL)	1.46 (±1.2)	1.44 (±1.1)	1.47 (±1.3)	0.46
Diabetes, n (%)	42 (40.7)	16 (15.5)	26 (25.2)	0.53
Hypertension, n (%)	48 (46.6)	22 (21.3)	26 (25.2)	0.43
History of kidney stones, n (%)	31(30.0)	9 (8.4)	22 (21.3)	0.08

Continuous variables are presented as mean ± SD. Categorical variables are presented as counts and percentages. Comparisons were performed using Student’s *t*-test for continuous variables and the chi-square test for categorical variables.

**Table 2:** Association between abscess size and the need for drainage.

Abscess Size	Drainage (n, %)	Conservative (n, %)	Total	RR (95% CI)
<50 mm	7 (14.9)	40 (85.1)	47	Reference
50 mm	53 (94.6)	3 (5.4)	56	6.35 (3.19–12.59)

Values are presented as counts and percentages. The RR with 95% CI was calculated to compare the probability of requiring drainage between groups.

**Table 3:** Patient characteristics according to abscess size.

Parameter	Overall (n = 103)	Abscess < 50 mm (n = 47)	Abscess 50 mm (n = 56)	*p*
Abscess size (mm)	60.4 (±41.1)	25.21 (±8.7)	90.05 (±33)	0.0001
Age (years)	60.53 (±16.3)	59.4 (±18.2)	61.48 (±14.8)	0.26
Gender F/M	59/44	26/21	33/23	0.86
Rural/Urban, n	62/41	23/24	39/17	0.032
Length of stay(days)	10.5 (±5.8)	10.4 (±5.4)	10.5 (±6)	0.47
WBC (/mmc)	13,893.3 (±6971.4)	14,012.34 (±8132.8)	13,793.4 (±5898.4)	0.437
Hb (g/dL)	10.65 (±2.1)	11.24 (±2.00)	10.1 (±2.1)	0.003
CRP (mg/dL)	142.1 (±100.7)	145.24 (±99.8)	139.4 (±102.3)	0.38
Serum creatinine (mg/dL)	1.46 (±1.2)	1.44 (±1.1)	1.5 (±1.3)	0.44
Diabetes, n (%)	42 (40.8)	18 (38.3)	24 (42.9)	0.63
Hypertension, n (%)	48 (46.6)	21 (44.7)	27 (48.2)	0.72
History of kidney stones, n (%)	31 (30.1)	17 (36.2)	14 (25)	0.21

Continuous variables are presented as mean ± SD. Categorical variables are presented as counts and percentages. Comparisons were performed using Student’s *t*-test and the chi-square test for categorical variables.

**Table 4:** Antibiotic resistance rates of isolated pathogens.

Antibiotic	Antibiotic Resistance Rates (%)
Amikacin	5.8
Amoxicilin	48.8
Ampicilin	79.5
Ceftriaxone	54.0
Cefuroxime	58.6
Ceftazidime	54.5
Ciprofloxacin	59.0
Gentamicin	38.8
Imipenem	17.5
Levofloxacin	56.6
Meropenem	11.1
Nitrofurantoin	26.6
Piperacillin/Tazobactam	6.2
TMP-SMX	52.0

Values represent the proportion of isolates resistant to each antibiotic.

**Table 5:** Spearman correlation between key variables.

Variable Pair	Spearman ρ	*p*-Value
Size vs. WBC	−0.0014	0.988555
Size vs. CRP	−0.0425	0.669649
WBC vs. CRP	0.5430	<0.001
Size vs. Hb	−0.2039	0.038831
Size vs. serum creatinine	−0.0886	0.373730

Spearman’s rank correlation coefficient (ρ) was used to assess the relationship between variables. *p*-values < 0.05 were considered statistically significant.

**Table 6:** Univariate logistic regression analysis.

Variable	OR	95% CI	*p*-Value
Abscess size	1.0796	1.0479–1.1123	0.00001
WBC	1.00	0.9999–1.0000	0.5888
CRP	1.00	0.9961–1.0039	0.9802
Haemoglobin	0.8244	0.6783–1.0020	0.0461
Age	1.010	0.9861–1.0347	0.4124
Serum creatinine	1.010	0.7302–1.3969	0.9522

Values are presented as OR with 95% CI. The dependent variable was the need for drainage.

**Table 7:** Multivariable regression analysis of factors associated with the need for drainage.

Variable	Model 1 OR (95% CI)	*p*-Value	Model 2 OR (95% CI)	*p*-Value
Abscess size (mm)	1.09 (1.06–1.13)	<0.001	1.09 (1.05–1.13)	<0.001
WBC (×10^3^/µL)	—	—	1.01 (0.97–1.05)	0.58
CRP (mg/dL)	—	—	1.00 (0.97–1.03)	0.81

Values are presented as OR with 95% CI. Model 1 includes abscess size only. Model 2 is a multivariable model adjusted for abscess size, WBC, and CRP. The dependent variable was the need for drainage.

**Table 8:** Predictive performance of models.

Metric	Size Only	Size + WBC + CRP
AUC	0.945	0.949
ΔAUC	—	0.004
*p*-value	—	0.74

AUC = area under the curve. ΔAUC represents the difference in predictive performance between models.

## Data Availability

The data presented in this study is available from the corresponding author upon reasonable request.

## Abbreviations

The following abbreviations are used in this manuscript:

AUC	Area Under the Curve
BUN	Blood Urea Nitrogen
CRP	C-Reactive Protein
CT	Computed Tomography
DMSA	Dimercaptosuccinic Acid
ESBL	Extended-Spectrum Beta-Lactamase
ESR	Erythrocyte Sedimentation Rate
Hb	Hemoglobin
HIV	Human Immunodeficiency Virus
MAR	Multiple Antibiotic Resistance
RDW	Red Cell Distribution Width
ROC	Receiver Operating Characteristic
UTI	Urinary Tract Infection
WBC	White Blood Cell

## References

[1] Huang L., Huang C., Yan Y., Sun L., Li H. (2022). Urinary Tract Infection Etiological Profiles and Antibiotic Resistance Patterns Varied Among Different Age Categories: A Retrospective Study From a Tertiary General Hospital During a 12-Year Period. Front. Microbiol..

[2] Jagadeesan S., Tripathi B.K., Patel P., Muthathal S. (2022). Urinary tract infection and Diabetes Mellitus-Etio-clinical pro-file and antibiogram: A North Indian perspective. J. Fam. Med. Prim. Care.

[3] López V.N., Ramos J.M., Meseguer V., Pérez Arellano J.L., Serrano R., Ordóñez M.A.G., Peralta G., Boix V., Pardo J., Conde A. (2009). Microbiology and outcome of iliopsoas abscess in 124 patients. Medicine.

[4] Dembry L.M., Andriole V.T. (1997). Renal and perirenal abscesses. Infect. Dis. Clin. N. Am..

[5] Hung C.H., Liou J.D., Yan M.Y., Chang C.C. (2007). Immediate percutaneous drainage compared with surgical drainage of renal abscess. Int. Urol. Nephrol..

[6] Fernandes R.C., Duarte P.D. (2002). Perinephric and renal abscesses in children: A study of three cases. Rev. Inst. Med. Trop. Sao Paulo.

[7] Yen D.H., Hu S.C., Tsai J., Kao W.F., Chern C.H., Wang L.M., Lee C.H. (1999). Renal abscess: Early diagnosis and treatment. Am. J. Emerg. Med..

[8] Wang I.K., Chen Y.M., Chen Y.C., Fang J.T., Hang C.C. (2003). Successful treatment of renal abscess with percutaneous needle aspiration in a diabetic patient with end stage renal disease undergoing hemodialysis. Ren. Fail..

[9] Mir R., Salari S., Najimi M., Rashki A. (2022). Determination of frequency, multiple antibiotic resistance index and resistotype of *Salmonella* spp. in chicken meat collected from southeast of Iran. Vet. Med. Sci..

[10] Kurdi Al-Dulaimi M., Abd Mutalib S., Abd Ghani M., Mohd Zaini N.A., Ariffin A.A. (2019). Multiple Antibiotic Resistance (MAR), Plasmid Profiles, and DNA Polymorphisms among Vibrio vulnificus Isolates. Antibiotics.

[11] Rubilotta E., Balzarro M., Lacola V., Sarti A., Porcaro A.B., Artibani W. (2014). Current clinical management of renal and perinephric abscesses: A literature review. Urologia.

[12] Bacha K., Miladi M., Ben Hassine L., Hajri M., Tanazaghti F., Ayed M. (2001). Aspects thérapeutiques des abcès du rein. A propos de 50 cas [Therapeutic aspects of renal abscess. Report of 50 cases]. Prog. Urol..

[13] Xia M., Liu J., Hong Y., An L., Xiong L., Huang X., Xu Q. (2020). Renal Abscess: Invasive Treatment or not. Res. Sq..

[14] Pesl M., Zámecník L., Pokuta P., Soukup V., Dvorácek J. (2004). Strategie lécby renálních abscesů [Treatment strategy for renal abscesses]. Rozhl. Chir..

[15] Joshi S., Leslie S.W., Desai D. (2025). Renal Abscess. [Updated 11 April 2025]. StatPearls [Internet].

[16] Coelho R.F., Schneider-Monteiro E.D., Mesquita J.L., Mazzucchi E., Marmo Lucon A., Srougi M. (2007). Renal and perinephric abscesses: Analysis of 65 consecutive cases. World J. Surg..

[17] Lee J., Hwang J.H., Yeom J.H., Lee S., Hwang J.H. (2024). Analysis of virulence profiles in clinical isolates of Klebsiella pneumoniae from renal abscesses: Clinical significance of hypervirulent isolates. Front. Cell. Infect. Microbiol..

[18] Eusebi L., Masino F., Bertolotto M., Montatore M., Sortino G., Pitoni L., Santarelli S., Galosi A.B., Guglielmi G. (2025). Contrast-Enhanced Ultrasound in the Evaluation and Management of Solid Renal Lesions Based on EFSUMB Guidelines. J. Med. Ultrason..

[19] Sosnowska-Sienkiewicz P., Bućko E., Mańkowski P. (2021). The Rare Case of Perirenal Abscess in a Child-Possible Mechanisms and Methods of Treatment: A Case Report and Literature Review. Medicina.

[20] Liu X.Q., Wang C.C., Liu Y.B., Liu K. (2016). Renal and perinephric abscesses in West China Hospital: 10-year retrospective-descriptive study. World J. Nephrol..

[21] Tejido Sánchez A., Jiménez de la Peña M.M., Duarte Ojeda J.M., Villacampa Aubá F., Martín Muñoz M.P., Lozano Ojeda F., Leiva Galvis O. (2000). Tratamiento percutáneo de los abscesos retroperitoneales [Percutaneous treatment of retroperitoneal abscesses]. Actas Urol. Esp..

[22] Magliano E., Grazioli V., Deflorio L., Leuci A.I., Mattina R., Romano P., Cocuzza C.E. (2012). Gender and age-dependent etiology of community-acquired urinary tract infections. Sci. World J..

[23] Zhang X., Xie Y., Huang G., Fu H. (2019). Analysis of 17 children with renal abscess. Int. J. Clin. Exp. Pathol..

[24] Rai R.S., Karan S.C., Kayastha A. (2007). Renal and Perinephric Abscesses Revisited. Med. J. Armed Forces India.

[25] El-Nahas A.R., Faisal R., Mohsen T., Al-Marhoon M.S., Abol-Enein H. (2010). What is the best drainage method for a perinephric abscess?. Int. Braz. J. Urol..

[26] Zhou Z., Gan F., Zhao K., Liu S., Xiao X., Li W., Han X., Wang W., Dong Z. (2025). A case of IgG4-related disease misdiagnosed as perirenal abscess: Case report and literature review. Medicine.

[27] Ivănuță M., Puia D., Cimpoeșu D.C., Ivănuță A.M., Bîcă O.D., Pricop C. (2024). Longitudinal Evaluation of Renal Function in Patients with Acquired Solitary Kidney-Urological Perspectives Post-Nephrectomy. J. Clin. Med..

[28] Stoian M., Azamfirei L., Stîngaciu A.C., Negulici L.-M., Văsieșiu A.M., Manea A., Stoian A. (2025). Early Diagnostic Markers and Risk Stratification in Sepsis: Prognostic Value of Neutrophil-to-Lymphocyte Ratio, Platelets, and the Carmeli Score. Biomedicines.

[29] Stoian M., Azamfirei L., Bandila S.R., Stoian A., Babă D.-F., Bănescu C. (2025). Circulating microRNAs and Plasma Gelsolin as Biomarkers of Sepsis: Molecular Insights and Prospects for Precision Medicine. Biomolecules.

[30] Canny S.P., Orozco S.L., Thulin N.K., Hamerman J.A. (2023). Immune Mechanisms in Inflammatory Anemia. Annu. Rev. Immunol..

[31] Lepanto M.S., Rosa L., Paesano R., Valenti P., Cutone A. (2019). Lactoferrin in Aseptic and Septic Inflammation. Molecules.

[32] Kulik-Rechberger B., Dubel M. (2024). Iron Deficiency, Iron Deficiency Anaemia and Anaemia of Inflammation—An Overview. Ann. Agric. Environ. Med..

[33] Chaparro C.M., Suchdev P.S. (2019). Anemia Epidemiology, Pathophysiology, and Etiology in Low- and Middle-Income Countries. Ann. N. Y. Acad. Sci..

